# Editorial Correspondence—the World’s Columbian Dental Congress

**Published:** 1893-09

**Authors:** 


### Editorial.

### EDITORIAL CORRESPONDENCE—THE WORLD'S COLUMBIAN DENTAL CONGRESS.

CHICAGO, August 19, 1893.

THIS is the last day of the long-expected and much-worked-for Congress, and now that it has closed its labors it is proper to give the impressions it has created, for more than this could not be given in a letter.

The number present, while large, has been by no means equal to general expectations, being, up to Friday, nine hundred and seventy-one Americans and one hundred and twenty foreigners.

The arrangements prepared by the Executive Committee, while judicious from an orderly point of view, have not been entirely satisfactory in other respects; indeed, have had the effect to measurably destroy interest in the proceedings.

The programme, as arranged, called for a general meeting of the Congress each day at 12 M. At this meeting one paper of supposed general interest was to be read. The discussion (?) was generally limited to one person, or cut off entirely at the option, apparently, of the presiding officer. At 2.30 P.M. the eight sections were called to order in different rooms, each section having the consideration of one subject. In the evening, lectures and lantern exhibits. The real work of the Congress was comprised between 2.30 and 5 o'clock. This, briefly, is an outline of the order of business.

The first day, Monday, the 14th, extended the time very considerably. Beginning at 10.30 A.M., the Congress was opened by prayer by Professor Taft, followed by a short address of welcome by President Bonney, of the World's Congress Auxiliary. The ceremony of introducing the officers of the World's Congress and foreign visitors then took place. In the case of the latter short responses were made.

The President then delivered his address, choosing for his subject the prominent historical periods in dentistry.

Very little can be said in commendation of this day's work. It was made entirely too long, the session ending at half-past two. It was tedious and in many respects unworthy the occasion.

The paper on Tuesday at 12 M. was, doubtless, a valuable one from a scientific stand-point, but the acoustic properties of the hall being bad, combined with the foreign accent of the reader, made it difficult to understand the thought of the writer.

The first experience with the work of the sections took place that afternoon. A brief examination of the rooms demonstrated quite conclusively that this plan of holding dental conventions could not be an entire success. This should have been foreseen. The natural feeling is while sitting in one room with twenty or thirty others, isolated from the balance of the Congress, that possibly the subject and paper under consideration may not be the most interesting of the series. This induced a restless feeling, and the constant stir of moving feet only added to the difficulty, and was not conducive to a scientific frame of mind.

Some of the sectious have been throughout the week largely attended, but in others it seemed difficult to secure auditors sufficient to give life to the proceedings.

The impossibility of dividing one's personality into eight sections left the feeling at the close of the day of having accomplished very little of real scientific value; at least such was the experience of the writer. Truth requires that this was not the experience of some fortunate ones, who reported exceedingly valuable sessions in some sections. It is to be hoped that the full report may show something worthy of the Congress, and for this all who desire to secure a correct idea of the work accomplished will be obliged to wait.

It is to be presumed that the possibility of great crowds led to this division, and the Executive Committee was probably fully justified in acting as they did; but as the expected thousands have not put in an appearance during the week, other arrangements could have been made far more satisfactory to those in attendance. The large hall with its galleries would have accommodated all. There is a magnetism in numbers which the section arrangement can never give.

Had the committee borne in mind the great success of the Oral Section of the International Medical Congress at Washington, D. C., they would never have consented to this subdivision.

The arrangements for the clinics were about as bad as they well could be. The room was not large enough, and combined with a low ceiling made an atmosphere unbearable. It is always difficult to make clinics satisfactory, and these were practically rendered valueless by the conditions and environments.

The government of the meetings has been in the highest degree autocratic. The person who had paid his ten or more dollars to become a member naturally indulged the expectation that he would have the privileges usually accorded to such. Those who entertained this idea must have been rudely shocked, as nothing of the kind was permitted.

Each member was provided with a *button*, and unless this was in its place he was not allowed to enter, a guard being placed at all doors. If a member made or attempted to make a motion in the general meeting he was immediately called to order, and informed that the Executive Committee had provided for all contingencies.

In a social sense the Congress has been a decided success, and if it have no other good result, this will amply justify its having been called. For the first and perhaps the last time persons widely separated have met and interchanged social courtesies as well as scientific opinions. In this respect it was the most remarkable gathering the writer has ever attended. There were several reunions of college graduates during the week, notably that of the Department of Dentistry of the University of Pennsylvania and also one by the Department of Dentistry of the University of Michigan.

The Women Dentists of the World held a meeting on Friday morning. It was largely attended by delegates from various States and by several from abroad. The session was an exceedingly interesting one, and as the first international gathering of women dentists, may be considered the most remarkable gathering resulting from this Congress. It marked a distinct epoch in the world's thought.

Whether the Congress has proved a scientific success it is difficult to determine at this writing, for the reasons given; but it certainly has had one good result, in drawing a large number of people to the last and, perhaps, the greatest of the world's efforts, the Exposition at Jackson Park. Its marvellous beauty and value from an educational stand-point must amply repay for the time and expense involved in getting here, even for those who may have encompassed half the earth to see it.

